# Laparoscopic Sleeve Gastrectomy With 75-mm Stapler Reloads: A Pilot Survey of Surgeon-Reported Workflow and Perceived Safety

**DOI:** 10.7759/cureus.117412

**Published:** 2026-10-03

**Authors:** Riwa Deghaim, Safwan Taha

**Affiliations:** 1 Medical Research, Independent Research, Beirut, LBN; 2 Surgery, The Metabolic and Bariatric Surgery Centre (COEMBS), Mediclinic Airport Road Hospital, Abu Dhabi, ARE

**Keywords:** bariatric surgery, operative workflow, staple-line junctions, stapler reload, surgeon survey, surgical ergonomics, surgical stapler

## Abstract

​​​​​​Introduction

Laparoscopic sleeve gastrectomy (LSG) requires multiple sequential stapler firings, creating junctions along the staple line. Extended-length 75 mm reloads can reduce the number of firings and junctions required, yet real-world surgeon experience with these devices in clinical practice remains largely undescribed. This evidence gap motivated the present study, which aimed to characterize surgeon-reported experience with 75 mm stapler reloads during LSG across workflow efficiency, staple-line quality, safety, cost, and adoption.

Methods

A cross-sectional anonymous web-based survey was distributed to bariatric surgeons in Latin America and the Middle East/Africa. Eligible participants had performed at least 10 LSG procedures using 75 mm reloads. Results are reported using descriptive statistics.

Results

Thirty-five of 60 invited surgeons completed the survey (35/60, 58.3% response rate). Most reported using three 75 mm cartridges (25/35, 71.4%) versus five standard cartridges (31/35, 88.6%), and 28/35 (80.0%) estimated two fewer staple-line junctions. Most estimated a shorter overall procedure duration. Reduced leak and bleeding risk were reported by 15/35 (42.9%) and 17/35 (48.6%), respectively; increases were perceived by 2/35 (5.7%) and 3/35 (8.6%). Staple-line formation was reported as improved by 24/35 (68.6%), unchanged by 8/35 (22.9%), and slightly disrupted by 3/35 (8.6%). Sixteen respondents (16/35, 45.7%) volunteered initial-antral positioning difficulty and hybrid 60/75 mm sequencing. Reduced fatigue was cited by 16/35 (45.7%), reduced cost by 31/35 (88.6%), and 30/35 (85.7%) would recommend the reloads.

Conclusions

Surgeons generally reported favorable workflow, safety, and cost perceptions with 75 mm reloads. Free-text responses identified staged 60/75 mm sequencing at the initial antral firing as a hypothesis-generating technical adaptation. Prospective studies using objective clinical and economic outcomes are warranted.

## Introduction

Laparoscopic sleeve gastrectomy (LSG) is the most commonly performed bariatric procedure worldwide [1]. LSG creates a long gastric staple line without an anastomosis. Each additional firing creates an overlap or junction along this staple line, making stapling technique and staple-line integrity central technical considerations [2]. Staple-line leak and hemorrhage remain the most serious complications of LSG; systematic review data report an overall leak rate of approximately 1.5% [3], and these complications are among the most frequent causes of perioperative morbidity and mortality in bariatric surgery. LSG typically requires five to six sequential stapler firings to complete the gastric transection [4,5]. This firing count carries measurable clinical consequences, as observational studies have linked a higher number of firings to increased intraoperative blood loss, postoperative bleeding, and prolonged hospital stay [5,6]. Furthermore, operative time has been independently associated with staple-line leak risk at one year [7]. Gastric wall thickness varies considerably along the sleeve axis, thicker at the antrum and thinner at the fundus, further complicating staple-line consistency and cartridge selection [8,9].

Taken together, these factors establish LSG as a procedure in which firing count, operative efficiency, and staple-line integrity are tightly interconnected. One modifiable strategy is to reduce the number of stapler firings required to complete gastric transection. Extended-length 75 mm laparoscopic reloads implement this strategy by covering a longer segment per firing and thereby reducing the expected number of firings and staple-line junctions. Although 75 mm laparoscopic reloads are commercially available from more than one manufacturer, they remain less widely represented than conventional 45 mm and 60 mm endoscopic systems. The evaluated cartridge combines a 75 mm reload length with a stepped cartridge face, graduated staple heights, and conventional B-form staple closure. B-form closure and graduated compression are established stapling principles rather than unique features of this product; the evidence gap concerns surgeon experience with their application in a 75 mm laparoscopic configuration. These cartridges are referred to hereafter as 75 mm reloads.

Despite the clinical plausibility of this approach, a critical evidence gap exists. Device-performance and outcomes studies can establish the technical efficacy of stapling systems under controlled conditions, but they do not capture how surgeons experience these devices in routine clinical practice, how they perceive workflow impact, whether they feel confident with staple-line integrity and reliability, what intraoperative difficulties they encounter, and how these perceptions influence their adoption decisions. For a less widely represented configuration such as the 75 mm laparoscopic reload, this surgeon-level usability evidence complements clinical outcome data by clarifying real-world adoption considerations and identifying meaningful targets for device refinement.

Methodologically, surgeon-perception surveys have characterized preferences for endocutter features such as articulation and jaw aperture [10], and anonymous web-based surveys have demonstrated the feasibility of describing bariatric surgical practice without patient-level information [11]. To our knowledge, no published study has evaluated real-world surgeon experience with 75 mm laparoscopic reloads - including perceived effects on firing count, staple-line quality, workflow, safety, cost, and operative decision-making. This pilot survey therefore aimed to characterize surgeon-reported experience with 75 mm stapler reloads in LSG across workflow efficiency, staple-line quality, technical performance, perceived safety, cost, and adoption.

## Materials and methods

Study design and reporting

We conducted a cross-sectional, descriptive, web-based survey of bariatric surgeons' experience with 75 mm stapler reloads in LSG. The reload evaluated by respondents was the XNY 75 mm endoscopic linear cutter reload (Changzhou XinNeng Yuan Medical Stapler Co., Ltd., Changzhou, China). According to manufacturer specifications, the reload is compatible with the manufacturer's manual and powered endoscopic linear-cutter handles. The survey evaluated the reload configuration rather than comparing handle actuation modes. Reporting followed the Strengthening the Reporting of Observational Studies in Epidemiology (STROBE) guidelines for cross-sectional studies [12] and the Checklist for Reporting Results of Internet E-Surveys (CHERRIES) recommendations for web-based surveys [13].

Survey administration and period

The questionnaire was created using Google Forms (Google LLC, Mountain View, California, USA) and distributed between July 2025 and March 2026 using a nonprobability, network-based sampling approach. Recruitment drew on the investigators’ professional surgical networks and additional professional contacts facilitated by XNY Medical. The anonymous survey link was circulated individually through email and professional messaging platforms. The survey was administered by the investigators; participation was voluntary, and no incentives were provided. XNY Medical had no access to individual responses and did not participate in data analysis or interpretation. Of the 60 bariatric surgeons invited, 35 completed the questionnaire (35/60, 58.3% response rate). The survey captured surgeons’ perceptions of cartridge utilization, staple-line junctions, staple-line formation, operative workflow and efficiency, technical performance, perceived safety, cost, and adoption (see Appendices).

Participants

Eligible respondents were bariatric surgeons who perform LSG and had performed at least 10 LSG procedures using the 75 mm stapler reloads. This minimum-experience threshold was applied to ensure that respondents had repeated hands-on experience with the device.

Instrument development and pre-testing

Survey items were drafted based on the study team's clinical experience and relevant stapling literature, refined for clarity by a bariatric surgeon and one researcher, and pilot-tested with three surgeons prior to launch. These three pilot responses were used only to refine the instrument and were excluded from the analysis; the final analysis set comprised the 35 subsequent respondents.

Survey quality and data completeness

The survey required approximately five to seven minutes to complete. Most items were required, though a small number of responses were incomplete for individual items, and percentages are reported against the number of respondents answering each item. Adaptive questioning and item randomization were not used. Responses were collected anonymously, and no account or device identifiers were retained; respondents were instructed to complete the survey only once. No personal identifiers, institutional identifiers, IP addresses, or location data were collected. All 35 submitted questionnaires were included in the analysis; item-specific denominators reflect missing responses. No data imputation was performed.

Outcomes and analysis

The primary objective was descriptive characterization of surgeons' perceptions across the predefined survey domains. Results are reported as counts and percentages. No hypothesis testing or inferential statistical analysis was planned or performed, given the pilot nature of the study, the small sample size, and the use of convenience sampling. Free-text responses were reviewed and grouped into recurring themes, which are reported as counts of respondents who volunteered each theme.

Ethics

This anonymous survey collected no patient-level, personally identifying, institutional, IP-address, or location data. The local ethics committee determined that formal evaluation was not required because the project was an anonymous descriptive questionnaire study involving healthcare professionals. Participation was voluntary, and submission of the completed questionnaire indicated consent to participate.

## Results

Thirty-five bariatric surgeons from Latin America and the Middle East/Africa completed the survey. Most respondents (28/35, 80.0%) reported more than 10 years of experience performing LSG, and 19/35 (54.3%) reported performing more than 20 LSG procedures per month (Table 1). Practice settings and primary surgical techniques are summarized in Table 1. Three respondents selected more than one primary technique category; these responses are reported as recorded.

**Table 1:** Respondent Characteristics (n=35) ^†^ Multiple selections allowed; percentages sum to >100%. ^‡^ Three respondents selected more than one technique category; responses are reported as recorded. LSG: laparoscopic sleeve gastrectomy

Characteristic	n (%)
Years performing LSG	
<3 years	1 (2.9)
3-5 years	3 (8.6)
6-10 years	3 (8.6)
>10 years	28 (80.0)
LSG cases per month	
<5	1 (2.9)
5-10	8 (22.9)
11-20	7 (20.0)
>20	19 (54.3)
Practice setting^†^	
Private hospital	31 (88.6)
Academic/teaching	9 (25.7)
Public/government	3 (8.6)
Community	2 (5.7)
Military	2 (5.7)
Primary technique^‡^	
Five port	15 (42.9)
Four port	13 (37.1)
Three port	8 (22.9)
Other (minilaparoscopic)	1 (2.9)

Workflow and efficiency

Compared with standard reloads, surgeons reported using fewer cartridges with 75 mm reloads. With standard reloads, 31/35 (88.6%) reported using five cartridges to complete LSG, whereas with 75 mm reloads, 25/35 (71.4%) reported using three cartridges and 7/35 (20.0%) reported using four. The majority reported fewer staple-line junctions, with 28/35 (80.0%) estimating a reduction of two junctions, 6/35 (17.1%) estimating one fewer junction, and 1/35 (2.9%) reporting no difference.

A majority (26/35, 74.3%) reported reduced cartridge-replacement time (24/35, 68.6% substantial; and 2/35, 5.7% slight), while 5/35 (14.3%) reported no impact and 4/35 (11.4%) reported an increase. Overall, 33/35 (94.3%) estimated a reduction in procedure duration (25/35, 71.4% substantial; and 8/35, 22.9% slight), with 2/35 (5.7%) reporting no impact. Estimated time savings per case were reported as 1-5 minutes by 19/35 respondents (54.3%) and 6-10 minutes by 8/35 (22.9%), with a smaller proportion estimating longer savings and the remainder reporting no time savings. Estimated operative time shifted toward shorter categories with 75 mm reloads: the proportion estimating 46-60 minutes fell from 15/35 (42.9%) with standard reloads to 10/35 (28.6%) with 75 mm reloads, while the proportion estimating 31-45 minutes increased from 11/35 (31.4%) to 14/35 (40.0%) (Table 2).

**Table 2:** Workflow and Efficiency Outcomes (n=35) - Category not applicable for standard reloads; no respondent used three cartridges with standard-length reloads.

Outcome	Standard reloads, n (%)	75-mm reloads, n (%)
Cartridges used		
Three cartridges	-	25 (71.4)
Four cartridges	4 (11.4)	7 (20.0)
Five cartridges	31 (88.6)	3 (8.6)
Estimated operative time		
≤15 min	1 (2.9)	1 (2.9)
16-30 min	8 (22.9)	9 (25.7)
31-45 min	11 (31.4)	14 (40.0)
46-60 min	15 (42.9)	10 (28.6)
61-90 min	0 (0.0)	1 (2.9)
Cartridge replacement time		
Slightly increases	-	4 (11.4)
No impact	-	5 (14.3)
Slightly reduces	-	2 (5.7)
Significantly reduces	-	24 (68.6)
Overall procedure duration		
No impact	-	2 (5.7)
Slightly reduces	-	8 (22.9)
Significantly reduces	-	25 (71.4)

Staple-line quality and perceived safety

Although the majority of respondents reported fewer junctions, staple-line formation assessments were not uniformly favorable. Most respondents (24/35, 68.6%) reported improved or more uniform formation, 8/35 (22.9%) reported no noticeable difference, and 3/35 (8.6%) reported slightly disrupted formation (Table 3). Regarding perceived risk, 15/35 (42.9%) reported reduced leak risk and 17/35 (48.6%) reduced bleeding risk; 2/35 (5.7%) perceived increased leak risk and 3/35 (8.6%) increased bleeding risk (Table 3). When asked about broader patient outcomes, 21/35 (60.0%) reported no noticeable difference, 9/35 (25.7%) fewer major complications, and 10/35 (28.6%) fewer minor complications; multiple selections were permitted, and no respondent selected an increase in complications. Because case denominators and observation periods were not collected, these responses represent surgeon perceptions and cannot establish complication rates. In the questionnaire, major complications referred to staple-line leak and bleeding, the two most serious complications of LSG [3], which were additionally captured through dedicated balanced-response items (Table 3); minor complications referred to lower-grade events not requiring reintervention. A small proportion (2/35, 5.7%) also perceived a reduction in length of hospital stay.

**Table 3:** Staple-Line Quality and Safety Outcomes With 75-mm Reloads (n=35)

Outcome	Reduced/improved, n (%)	No difference, n (%)	Increased/disrupted, n (%)
Staple-line formation quality			
More uniform formation	15 (42.9)	-	-
Slightly improved formation	9 (25.7)	-	-
No noticeable difference	-	8 (22.9)	-
Slightly disrupted formation	-	-	3 (8.6)
Perceived leak risk	15 (42.9)	18 (51.4)	2 (5.7)
Perceived bleeding risk	17 (48.6)	15 (42.9)	3 (8.6)

When asked about broader patient outcomes, 60.0% reported no noticeable difference, 25.7% perceived fewer major complications, and 28.6% perceived fewer minor complications. Multiple selections were permitted, and no respondent selected an increase in complications. Because case denominators and observation periods were not collected, these responses represent surgeons’ perceptions and cannot establish complication rates. In the questionnaire, major complications referred to staple-line leak and bleeding, which were also captured through dedicated balanced-response items in Table 3. Minor complications referred to lower-grade events not requiring reintervention. A small proportion (5.7%) also perceived a reduction in hospital length of stay.

Technical performance

Regarding ease of positioning, 5/35 respondents (14.3%) found the 75 mm reload easier to position, 10/35 (28.6%) reported greater difficulty, and 20/35 (57.1%) reported no difference. Tissue slippage was reported as unchanged by 32/35 respondents (91.4%), with 2/35 (5.7%) reporting increased slippage and 1/35 (2.9%) reporting reduced slippage. For the additional-benefits item, which permitted multiple selections, reduced surgeon fatigue was the most frequently reported benefit (16/35, 45.7%), followed by reduced technical complexity (10/35, 28.6%), improved staple-line orientation and visualization (10/35, 28.6%), and greater ease of use in difficult anatomy (5/35, 14.3%); 11/35 (31.4%) reported no additional benefits.

First-firing difficulty and hybrid sequencing

In free-text responses, 16/35 respondents (45.7%) described a consistent pattern centered on the first firing. These surgeons identified the initial antral firing as the most technically demanding and most critical step, reported greater difficulty positioning the 75 mm reload at this point, and consequently began transection with a 60 mm reload before transitioning to 75 mm reloads for the remaining firings. The same subgroup reported progressing to exclusive 75 mm use once familiarity with the device was established. No closed-response item specifically addressed cartridge-sequencing strategy; these observations were drawn from open-ended comments and therefore represent volunteered rather than systematically elicited responses. The number volunteering first-firing difficulty in free text (16) exceeds the number reporting greater difficulty on the closed positioning item (10); this may reflect that the two items addressed different aspects: the closed item captured overall ease of positioning across the sleeve, whereas the free-text comments concerned the initial antral firing specifically, so a respondent could report acceptable overall positioning while still describing the first firing as the most demanding step.

Cost and adoption

Cost per procedure was reported as reduced by 31/35 respondents (88.6%; 16/35, 45.7% slightly; and 15/35, 42.9% substantially), while 3/35 (8.6%) reported no difference and 1/35 (2.9%) a slight increase. When asked to assess the overall value of 75 mm reloads considering both clinical benefits and cost, 8/35 (22.9%) reported that benefits outweighed costs, 11/35 (31.4%) that costs and benefits were approximately equal, 3/35 (8.6%) that costs outweighed benefits, and 13/35 (37.1%) that they were unable to assess value. A total of 30/35 (85.7%) indicated that they would recommend 75 mm reloads to colleagues.

## Discussion

Most respondents reported workflow benefits with 75 mm reloads, including fewer cartridges, fewer staple-line junctions, and shorter cartridge-replacement time. The survey also identified nuances in staple-line formation, positioning, and cost-value judgments that efficiency measures alone did not capture. These findings describe surgeons’ perceptions rather than prospectively measured clinical outcomes. Accordingly, the reported differences in workflow, safety, and cost should be interpreted as experiential observations and not as demonstrated comparative clinical or economic benefits.

These findings extend prior surgeon surveys. Gutierrez et al. identified articulation and jaw aperture as prioritized endocutter features across specialties [10]; our data identify reload length and its relationship to firing count as an additional usability dimension specific to LSG. A nationwide survey of Polish bariatric centers documented technical variability in reinforcement and cartridge selection [14]; the present findings add reload length as another technical parameter linked by respondents to workflow and staple-line perceptions. Reload length may therefore warrant consideration alongside staple height, reinforcement technique, and bougie size when studying LSG standardization.

The survey is consistent with mechanical evidence that reducing firings or eliminating junctions may improve staple-line consistency [15]. Clinical studies of 23-cm single-fire sleeve staplers have reported favorable operative-time profiles and low complication rates [16,17], offering indirect but non-transferable support for the general principle that fewer firings may benefit workflow and staple-line integrity. Most respondents reported a reduction from five to three cartridges and approximately two fewer junctions. This pattern is plausible given that the LSG staple line is approximately 200-230 mm and commonly requires several firings with standard 60 mm cartridges [8,9]. The reported operative-time reduction is also consistent with the expected effect of fewer firings and with learning-curve evidence linking procedural experience to shorter operative time [4]. In a large observational analysis, each additional 10 minutes of LSG operative time was independently associated with greater one-year staple-line leak risk [7]; this supports prospective measurement of operative time without implying that the present survey demonstrated a clinical benefit.

Despite the reported reduction in junction frequency, staple-line formation assessments were mixed: 3/35 respondents (8.6%) reported slight disruption, whereas 24/35 (68.6%) reported improved or more uniform formation. Most respondents nevertheless reported unchanged or reduced perceived leak and bleeding risk. The closed-response item did not identify where any formation disruption occurred, so that signal cannot be localized. The free-text comments separately focused on the initial antral firing. The antrum is the thickest region of the stomach, and its thickness varies considerably between patients; reported associations with BMI and sex are inconsistent [8,9]. A longer jaw may also change positioning and compression demands at this first firing, providing a plausible reason to evaluate this step directly rather than infer causation from the survey.

Consistent with this anatomical challenge, nearly half of respondents (16/35, 45.7%) volunteered in free-text responses that positioning the 75 mm reload at the initial antral firing was the most demanding step. These respondents described beginning transection with a 60 mm reload and reserving 75 mm reloads for the subsequent, thinner segments of the sleeve, with some progressing to exclusive 75 mm use as familiarity increased. Individualizing the antral cartridge to tissue thickness is established practice, with technique guidance recommending thicker cartridges at the antrum to avoid tissue crushing and staple-line failure [8,9]. Earlier clinical experience described flexible 55/75 mm stapling systems across mixed laparoscopic and conventional procedures, including LSG [18], but focused evidence on contemporary 75 mm laparoscopic reloads remains limited. The recurrence of this pattern in volunteered comments suggests a potentially meaningful usability signal. However, because sequencing was not assessed through a prespecified closed-response item, its frequency and comparative performance cannot be established from the present survey. Accordingly, the staged use of a 60 mm reload for the initial antral firing followed by 75 mm reloads appears to represent a novel, practice-derived technical adaptation. It provides a focused hypothesis for prospective evaluation, but the present data do not establish it as a preferred technique.

The concentration of these reports within a defined subgroup, together with the minority reporting disrupted formation, may suggest a learning effect associated with extended-length reloads. Staple-line complications after LSG are multifactorial and influenced by patient, technical, and device-related factors [3], and the relationship between reload length, formation quality, and clinical risk remains incompletely understood. This finding underscores the importance of evaluating staple-line integrity as an independent endpoint in future prospective studies, and of comparing the hybrid 60/75 mm sequence against exclusive 75 mm use, rather than inferring formation quality solely from junction count.

Reduced surgeon fatigue was the most frequently cited additional benefit (16/35, 45.7%). This is relevant because laparoscopic instrument use imposes measurable ergonomic and muscular demands [19], and fatigue or excessive workload was cited as a contributing factor in roughly one-third of incidents in an interview-based analysis of surgical errors [20]. Fewer firings and less repetitive instrument manipulation may reduce this handling burden, offering a plausible mechanism - though objective measurement of operative fatigue remains difficult and warrants validated cognitive-workload and ergonomic assessment in future work.

The cost data revealed a telling conflict. Although 31/35 respondents (88.6%) reported reduced per-procedure cost, only 8/35 (22.9%) concluded overall benefits outweighed cost, and over a third could not assess overall value. This is relevant given that stapling devices are the single largest cost contributor in LSG, accounting for approximately 27.7% of procedural cost [21]; a reduction from five to three cartridges is therefore a plausibly meaningful saving, although its magnitude will depend on institutional procurement and local pricing. The gap between perceived savings and cautious overall-value judgments suggests surgeons acknowledge economics while withholding full endorsement absent objective outcome data. The 30/35 (85.7%) recommendation rate indicates experiential value, but formal cost-benefit analysis remains necessary.

The scientific contribution of this survey does not lie in B-form staple closure or graduated compression, which are established endoscopic-stapling principles, but in characterizing surgeon experience with these features in a 75 mm laparoscopic configuration during LSG. The reported reductions in cartridge use, junctions, and estimated operative time confirm expected mechanical effects. More distinctive findings were reduced surgeon fatigue, difficulty positioning the longer jaw at the initial antral firing, the surgeon-derived hybrid 60/75 mm sequence, and cautious value judgments despite perceived cost savings. These human-factors and adoption findings are not available from bench testing and, to our knowledge, have not been reported together for 75 mm laparoscopic reloads. They constitute the study's principal novelty and provide specific hypotheses for product refinement and prospective comparison.

Study strengths

The study used balanced bidirectional response scales, required at least 10 prior LSG procedures with the evaluated reload, and compared each domain with surgeons' standard reload practice. The 35/60 (58.3%) response rate compares favorably with published response rates for surgical healthcare-professional surveys [22]. Anonymous participation and inclusion of surgeons from two regions and several practice settings enabled reporting of both favorable and unfavorable experiences. Combining closed items with free-text responses captured not only expected workflow effects but also specific human-factors observations - particularly initial-firing difficulty and hybrid sequencing - that can directly inform product refinement and prospective study design.

Limitations

Several considerations frame the interpretation of this pilot survey. Network-based recruitment and voluntary participation may have introduced selection and response bias, while the sample of 35 respondents from two regions, predominantly highly experienced surgeons working in private hospitals, may limit generalizability. Because the outcomes reflect surgeon perceptions, the findings are best viewed as real-world usability and adoption signals that complement objective clinical and economic evidence rather than establish comparative benefit. The initial-firing and hybrid-sequencing themes arose from volunteered free-text comments rather than a prespecified closed-response item; consequently, they remain hypothesis-generating, although their recurrence identifies a focused question for prospective investigation. Future studies should compare 75 mm and 60 mm reloads, including exclusive and hybrid sequencing, using objective measures of firing count, operative time, staple-line integrity, intraoperative intervention, cost, leak, and bleeding.

## Conclusions

In this exploratory survey, experienced bariatric surgeons reported favorable perceptions of workflow, safety, and cost with 75 mm laparoscopic reloads, most often alongside fewer cartridges and staple-line junctions. The initial antral firing emerged as the principal usability challenge. Free-text responses also identified a staged 60/75 mm approach as a potentially novel, practice-derived hypothesis for prospective evaluation rather than an established preferred technique. These findings do not demonstrate comparative clinical or economic benefit but identify specific targets for device refinement and objective prospective investigation.

## Appendices

Clinical evaluation of 75 mm endoscopic reloads in sleeve gastrectomy

Thank you for participating in this anonymous survey evaluating surgeons’ experience with 75 mm endoscopic reloads in sleeve gastrectomy. The survey examines operative workflow, perceived safety, technical performance, and cost considerations. We appreciate your time and expertise.

The 75 mm reload evaluated in this survey was manufactured by Changzhou XinNeng Yuan Medical Stapler Co., Ltd. (XNY Medical, Changzhou, China).

Surgeon Profile

Q1. Years performing laparoscopic sleeve gastrectomy

Select one:

○ Less than 3 years

○ 3-5 years

○ 6-10 years

○ More than 10 years

Q2. Approximate number of sleeve gastrectomy procedures performed monthly

Select one:

○ Less than 5

○ 5-10

○ 11-20

○ More than 20

Q3. Practice setting

Select all that apply:

□ Academic/teaching hospital

□ Private hospital

□ Public/government hospital

□ Military hospital

□ Community hospital

□ Other

Q4. If you selected “Other” as the practice setting, please specify:

Q5. Primary sleeve gastrectomy technique used

Select all that apply:

□ 5-port technique

□ 4-port technique

□ 3-port technique

□ Single-incision laparoscopic surgery

□ Robotic-assisted

□ Other

Q6. If you selected “Other,” please specify:

Operative Time Assessment

Please evaluate how 75 mm reloads affect operative time during sleeve gastrectomy. Estimates based on your experience may be provided.

Q7. When using standard-length reloads (30-60 mm), what is your average operative time for sleeve gastrectomy?

Select one:

○ 15 minutes or less

○ 16-30 minutes

○ 31-45 minutes

○ 46-60 minutes

○ 61-90 minutes

○ More than 90 minutes

Q8. When using 75 mm reloads, what is your average operative time for sleeve gastrectomy?

Select one:

○ 15 minutes or less

○ 16-30 minutes

○ 31-45 minutes

○ 46-60 minutes

○ 61-90 minutes

○ More than 90 minutes

Q9. Estimated time saved per procedure when using 75 mm reloads

Select one:

○ No time saved

○ 1-5 minutes

○ 6-10 minutes

○ 11-15 minutes

○ More than 15 minutes

Q10. How does the 75 mm reload affect the time spent replacing cartridges?

Select one:

○ Significantly increases time

○ Slightly increases time

○ No impact

○ Slightly reduces time

○ Significantly reduces time

Q11. How does the 75 mm reload affect the overall procedure duration?

Select one:

○ Significantly increases time

○ Slightly increases time

○ No impact

○ Slightly reduces time

○ Significantly reduces time

Staple-Line Junctions and Patient Safety

Please evaluate how 75 mm reloads affect staple-line junctions and perceived patient-safety outcomes.

Q12. Average number of staple cartridges used with standard reloads (30-60 mm) for complete sleeve construction

Select one:

○ 3 or fewer

○ 4

○ 5

○ 6

○ 7 or more

Q13. Average number of staple cartridges used with 75 mm reloads for complete sleeve construction

Select one:

○ 2 or fewer

○ 3

○ 4

○ 5

○ 6 or more

Q14. In your experience, how do 75 mm reloads affect the number of staple-line junctions required in sleeve gastrectomy compared with standard reloads?

Select one:

○ Require more junctions

○ No difference in the number of junctions

○ Reduce by approximately 1 junction

○ Reduce by approximately 2 junctions

○ Reduce by 3 or more junctions

Q15. How does the use of 75 mm reloads affect staple-line formation compared with standard 30-60 mm reloads?

Select one:

○ More uniform staple-line formation

○ Slightly improved staple-line formation

○ No noticeable difference

○ Slightly disrupted staple-line formation

○ Significantly disrupted staple-line formation

Q16. How do 75 mm reloads affect the risk of staple-line leaks compared with standard reloads?

Select one:

○ Significantly increase risk

○ Slightly increase risk

○ No difference in risk

○ Slightly reduce risk

○ Significantly reduce risk

Q17. How do 75 mm reloads affect the risk of staple-line bleeding compared with standard reloads?

Select one:

○ Significantly increase risk

○ Slightly increase risk

○ No difference in risk

○ Slightly reduce risk

○ Significantly reduce risk

Q18. Which patient outcomes, if any, are affected when using 75 mm reloads compared with standard reloads?

Select all that apply:

Major complications were defined as staple-line leak and bleeding. Minor complications were defined as lower-grade events not requiring reintervention.

□ Reduced length of hospital stay

□ Increased length of hospital stay

□ Faster resumption of oral intake

□ Delayed resumption of oral intake

□ Lower postoperative pain scores

□ Higher postoperative pain scores

□ Improved wound healing

□ Delayed wound healing

□ Fewer major complications

□ More major complications

□ Fewer minor complications

□ More minor complications

□ No noticeable differences in outcomes

Cost Efficiency

Please evaluate the economic impact of using 75 mm reloads. If exact cost information is unavailable, provide your best professional estimate based on your experience.

Q19. How do 75 mm reloads affect the overall cost per procedure?

Select one:

○ Significantly increase costs

○ Slightly increase costs

○ No significant cost difference

○ Slightly reduce costs

○ Significantly reduce costs

○ Unable to assess

Q20. Considering both the clinical benefits and costs, how would you assess the overall value of 75 mm reloads?

Select one:

○ The costs outweigh the benefits

○ The costs and benefits are approximately equal

○ The benefits outweigh the costs

○ Unable to assess

Technical Performance

Q21. How does the 75 mm reload compare with standard reloads in terms of ease of positioning during sleeve gastrectomy?

Select one:

○ Much more difficult to position

○ Somewhat more difficult to position

○ Similar ease of positioning

○ Somewhat easier to position

○ Much easier to position

Q22. Does using 75 mm reloads affect tissue compression compared with standard 30-60 mm reloads?

Select one:

○ Significant increase in tissue slippage

○ Slight increase in tissue slippage

○ No noticeable difference

○ Slight reduction in tissue slippage

○ Significant reduction in tissue slippage

Q23. Have you observed any additional benefits when using 75 mm reloads?

Select all that apply:

□ Improved staple-line orientation and visualization

□ Reduced technical complexity

□ Greater ease of use in difficult anatomy

□ Reduced surgeon fatigue

□ Other

□ No additional benefits observed

Q24. If you selected “Other,” please specify:

Q25. Would you recommend the 75 mm reload to colleagues for sleeve gastrectomy procedures?

Select one:

○ Yes

○ No

○ Uncertain 
